# Regulation of Aquaporin Prip Expression and Its Physiological Function in *Rhyzopertha dominica* (Coleoptera: Bostrichidae)

**DOI:** 10.3390/insects14010070

**Published:** 2023-01-11

**Authors:** Lan-Pin Tan, Mei-Er Chen

**Affiliations:** Department of Entomology, National Chung-Hsing University, Taichung City 40227, Taiwan

**Keywords:** *Rhyzopertha dominica*, aquaporin prip, transcriptional expression, ovary, RNAi

## Abstract

**Simple Summary:**

*Rhyzopertha dominica* Prip (*RdPrip*) cDNA was cloned and the developmental and tissue profiles of *RdPrip* transcription levels were determined using qPCR. *RdPrip* was highly transcribed in female adults and ovaries. This suggests that *RdPrip* plays a vital role in female reproduction. Knockdown of *RdPrip* in female adults through feeding of ds*RdPrip* RNA significantly decreased the hatching rate of eggs laid by the females, further decreasing the number of offspring.

**Abstract:**

*Rhyzopertha dominica* Prip (*RdPrip*) cDNA was cloned (GenBank accession no. OK318454), and the encoded 276-amino-acid protein indicated the typical aquaporin structure, including six transmembrane regions and two NPA motifs. The developmental and tissue profiles of *RdPrip* transcription were determined. *RdPrip* was highly transcribed in female adults, followed by larvae, pupae, and male adults. The transcriptional expression levels of *RdPrip* were significantly high in the ovary and hindgut (including cryptonephridial systems) compared with the foregut, testis, midgut, and Malpighian tubules. Knockdown of *RdPrip* in female adults did not decrease fecundity, but significantly decreased the hatching rate of eggs laid by the females. The results suggest that *RdPrip* functions in embryonic development, not in egg formation. In addition, the transcriptional expression level of *RdPrip* was lower in the spinosad-resistant strain than in the susceptible one, and the resistant strain produced fewer progeny than the susceptible strain did. These studies support the functional role of *RdPrip* in female reproduction. The absence of significant mortality reduction in the *R. dominica* exposed to spinosad after *RdPrip* RNAi suggests that other aquaporins that were not knocked down may exist for the excretion of metabolized pesticides.

## 1. Introduction

Water plays a vital role in homeostasis. Nutrients, metabolites, hormones, and many other molecules are all dissolved in water for cell exchange. Although water can simply diffuse into a cell, rapid water movement across cell membranes is achieved by aquaporins [1]. Aquaporins are proteins located at the cell membrane to transport water and other small solutes. The insect aquaporin superfamily contains five main subfamilies: (1) *Drosophila* integral protein (Drip), (2) *Pyrocoelia rufa* integral protein (Prip), (3) Glyceroporin (Glp), (4) big brain (Bib), and (5) others [2]. Among them, Drip and Prip are water-selective, and they mainly transport water molecules [3,4,5].

Aquaporins play various physiological roles. Drip and Prip play crucial roles in the diuresis of blood-feeding and phloem-sucking insects [4,6,7,8,9]. Increased excretion rate by aquaporins is a mechanism by which insects develop resistance to insecticides [10,11]. In addition to excretion, aquaporins also regulate reproduction [12,13,14,15] and are used by many insects to combat environmental stresses, such as low temperatures and drought [16,17,18,19].

*Rhyzopertha dominica*, the lesser grain borer, lives in barns worldwide and feeds on dry grains, including wheat, barley, oats, sorghum, maize, peanuts, and paddy rice. It is a primary pest that damages grains, leading to an increased likelihood of secondary pest outbreaks and thus the worsening of economic loss. *R. dominica* can also harm nuts, woody plants, and wood products [20,21]. The living environments of *R. dominica* lack water, and the water content in food is low. It appears that water homeostasis is extremely important for *R. dominica*. Its excretory system is a special cryptonephridial system in which the blind ends of the Malpighian tubules are closely associated with the rectum and covered by the perinephric membrane [22]. Cryptonephridial systems greatly improve water reabsorption in the rectum. Considering the adaptation of *R. dominica* to dry environments without drinking water, it is thought-provoking to explore the functions of aquaporins in *R. dominica*.

In this study, full-length aquaporin cDNA, *RdPrip*, was cloned, and the transcriptional profiles of developmental stages and tissues were determined. The functional study was performed using RNAi. Control of *R. dominica* relies primarily on chemical insecticides. Disruption of insect water homeostasis may influence the excretion of metabolized insecticides. Therefore, the role of *RdPrip* in spinosad-resistant *R. dominica* was investigated for developing a strategy to control *R. dominca*.

## 2. Materials and Methods

### 2.1. Insects R. dominica

The susceptible strain was maintained in the laboratory for more than two years without exposure to any pesticides. The spinosad-resistant strain was maintained by selection about every two months by using a modified grain application method described elsewhere [23]. The spinosad (ENTRUST 80 WP (GF-733), 80% spinosad) was provided by Dow AgroSciences LLC (Indianapolis, IN, USA). Both strains were fed on wheat flakes and maintained in glass jars at 28 °C, 30 ± 1% RH, under a 12 h: 12 h photoperiod.

### 2.2. cDNA Cloning of RdPrip by RT-PCR and RACE, and Sequence Analysis

The templates for the reverse transcription reaction were obtained from 1 µg of total RNA from the susceptible *R. dominica* by using the GeneRacer Kit (Thermal, Carlsbad, CA, USA) following the manufacturer’s instructions. A polymerase chain reaction was applied with degenerate primers, RdDripdf and RdDripdr (Table 1), which were designed based on the consensus sequences of aquaporin drip from *Anomala cuprea* [24] (accession number: AB741517), *Bombyx mori* [25] (accession number: AB178640), *Culex quinquefasciatus* (accession number: XM_001865697), and *Rhodnius prolixus* [26] (accession number: HQ711952). The PCR product (394 bp) was cloned into a pGEM-T Easy vector (Promega, Madison, WI, USA). The cDNA fragment was sequenced using an ABI PRISM Big Dye Terminator Cycle Sequencing Core kit with AmpliTaq DNA polymerase (ABI, Foster City, CA, USA) and the sequences were obtained from Tri-I Biotech (Taipei, Taiwan).

The 5′ and 3′ ends of the putative *R. dominica* aquaporin cDNA were produced using the GeneRacer Kit (Thermal). Specific primers were designed based on the sequence of the 394-bp fragment. For initial 5′ RACE PCR, the primer pair was RdDrip450-2 (Table 1) and the GeneRacer 5′ primer (Thermal), and for the nested PCR, the primer pair was RdDrip450-4 and the GeneRacer nested 5′ primer (Thermal). For the initial 3′ RACE PCR, the primer pair was RdDrip450-1 (Table 1) and the GeneRacer 3′ primer (Thermal), and for the nested PCR, the primer pair was RdDrip450-3 and the GeneRacer nested 3′ primer (Thermal). The PCR products were purified, cloned, sequenced, and encompassed the full-length *R. dominica* aquaporin cDNA sequence. To avoid errors in the assembly process, specific sense and antisense primers, RdPrip1008-1 and RdPrip1008-2, respectively, were designed in the 5′ and 3′ untranslated regions to perform PCR to amplify the entire open reading frame in one piece of DNA. The DNA was purified, cloned, and sequenced.

Prediction of transmembrane regions was performed with DeepTMHMM [27] (https://dtu.biolib.com/DeepTMHMM, accessed on 7 October 2022). Percentage identity and similarity were calculated with the Needle Pairwise Sequence Alignment of EMBL-EBI [28] (https://www.ebi.ac.uk/Tools/psa/emboss_needle/, accessed on 7 October 2022).

### 2.3. Analysis of the Developmental and Tissue Expression Profiles of RdPrip by Real-Time Quantitative RT-PCR

Developmental and tissue expression profiles of *RdPrip* transcription were analyzed using real-time quantitative RT-PCR (qPCR). Total RNA (0.5 μg) from larvae, pupae, and female and male adults was reverse transcribed into cDNA by using SuperScript III Frist-Strand Synthesis SuperMix (Thermal) for qRT-PCR, according to the manufacturer’s instructions. Total RNA (0.35 μg) from the adult foregut, midgut, hindgut (including cryptonephridial systems), Malpighian tubules, testes, and ovaries was similarly reverse transcribed into cDNA. The *18s* gene was used as the reference gene. The primers used in qPCR were RdPrip qPCR-1 and RdPrip qPCR-2 for the target gene, and Rd-18s qPCR-01 and Rd-18s qPCR-02 for the reference gene (Table 1). The qPCR reactions were performed using the CFX Connect Real-Time PCR System (Bio-Rad, Hercules, CA, USA) with iQ SYBR Green SuperMix (Bio-Rad). The PCR parameters for both target and reference genes were 95 °C for 3 min, 40 cycles of 95 °C for 10 s, and 60 °C for 30 s. A melting curve analysis was performed for each test to determine the specificity of the amplification. Three independent biological replicates of each sample and three technical repeats of each biological replicate were performed.

The qPCR data were collected, and the 2^−ΔΔ*CT*^ method was applied for quantitative data analyses. The qPCR data were analyzed using ANOVA followed by Tukey’s multiple comparison test.

### 2.4. Functional Studies of RdPrip through RNA Interference

The ds*RdPrip* RNA was synthesized using the MEGAscript RNAi Kit (Thermo), following the manufacturer’s instructions. The plasmid containing the cDNA of *RdPrip* or *Egfp* (control) served as templates for PCR amplification. The PCR primers, RdPripdsRNA-03, and RdPripdsRNA-04, with a T7 promoter sequence at the 5′ end and a gene-specific part for *RdPrip* are listed in Table 1. For the feeding treatment, 10 mated female adults of similar age were placed into one well of a 24-well plate containing 0.2 g of dried wheat flakes soaked with 35 μg of dsRNA in 300 μL of nuclease-free water. The plate was maintained at 28 °C for 3 days. Subsequently, the females were removed into another 24-well plate with egg collection paper for laying eggs. After 2 days of egg collection, the egg collection papers were removed into a Petri dish (9 cm in diameter) for hatching. There were six replicates in each treatment. *RdPrip* expression was determined using qPCR. All of the data were analyzed by ANOVA, followed by Tukey’s multiple comparison test.

### 2.5. Association of RdPrip Functions with Spinosad Resistance in R. dominica

To observe the differential expression of *RdPrip* between the susceptible and spinosad-resistant strains, 1 μg of total RNA was collected from adults of both susceptible and resistant strains for synthesizing the first-stand cDNA. qPCR reactions were performed using the iCycler iQ5 system (Bio-Rad) with iQ SYBR Green SuperMix (Bio-Rad). The qPCR programs were described earlier. A *t*-test was used to analyze the qPCR data.

To determine the progeny number of the two strains, 16 pairs of each strain were placed into a glass cylindrical cup (15.5 cm in height and 7.8 cm in diameter) filled with 80 g of wheat flakes as one group. Three groups of each strain were set up. A total of six cups were maintained at 28 °C, 30 ± 1% RH, and under a 12 h:12 h photoperiod for 45 days before assessment. The number of adults was counted, and 32 adults were designated as the parents. The number of first-generation adults between the two strains was compared using a *t*-test.

To detect the mortality of *R. dominica* exposed to spinosad after knocking down *RdPrip*, 20 adults were fed on wheat flakes soaked with water, ds*Egfp* RNA, and ds*RdPrip* RNA, as described earlier. There were five replicates for each treatment. After 3 days of feeding on dsRNA, the susceptibility of these adults to spinosad (4 μg/mL) was determined using a modified grain application method [23]. The mortality was calculated after 24 h of spinosad treatments. The mortality with the three treatments was analyzed using ANOVA followed by Tukey’s multiple comparison test.

*p* < 0.05 was set as statistically significant for all of the analyses in this study.

## 3. Results

### 3.1. cDNA Cloning and Sequence Analyses

*Rhyzopertha dominica* aquaporin prip (*RdPrip*) cDNA (GenBank accession no. OK318454) was cloned using RT-PCR and RACE. Our original intent was to clone the drip cDNA of the lesser grain borer. Therefore, the degenerate primers were designed based on the conserved transmembrane region of the four insect *drip*s. The first 394-bp PCR product appeared as an aquaporin. However, the 1008-bp full-length cDNA encoded a prip. The cloning of a different aquaporin occurred likely because the transmembrane and NPA regions of aquaporins are highly conserved [29,30]. This cloning was conducted before the *R. dominica* genome was released [31]. The aforementioned situation is less likely to occur with the genome.

**Figure 1:**
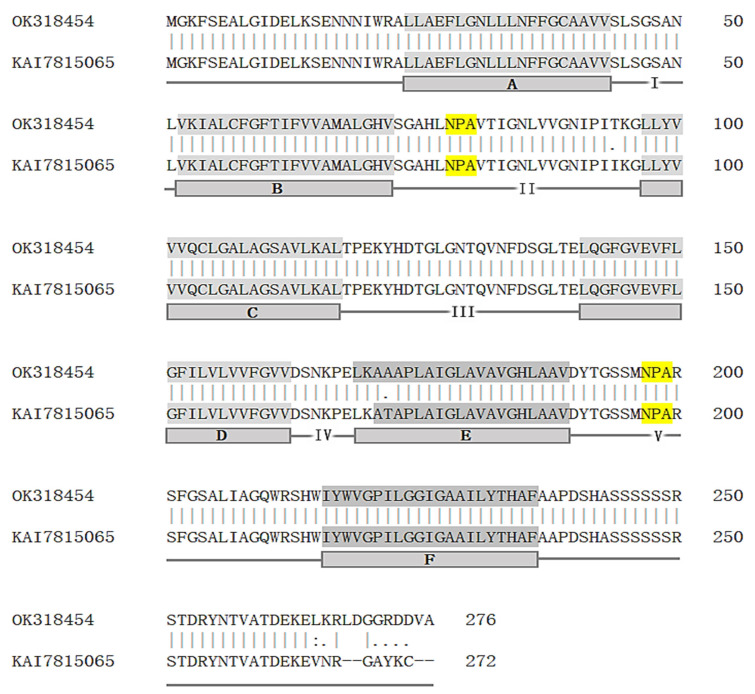
The alignment of amino acid sequences of *R. dominica* prip from one cloned from this study (Accession no. OK318454) and the other from the genome (Accession no. KAI7815065). The six transmembrane regions (**A**–**F**) are indicated by a gray background. The two NPA (Asn–Pro–Ala) motifs located on the second and fifth loops are marked in yellow.

The 1008-bp cDNA sequence contains an 831-bp open reading frame encoding a 276-amino-acid protein with six predicted transmembrane domains (TMs) and five loops adjacent to the TMs. Two highly conserved NPA (Asn–Pro–Ala) sequences of aquaporins were located in loops II and V (Figure 1). The amino acid sequence of *RdPrip* from this study shared 95.7% identity and displayed 96.0% similarity with that of prip from the *R. dominica* genome. The greatest difference was in the C-terminus (Figure 1).

### 3.2. Expression Profiles of RdPrip

**Figure 2:**
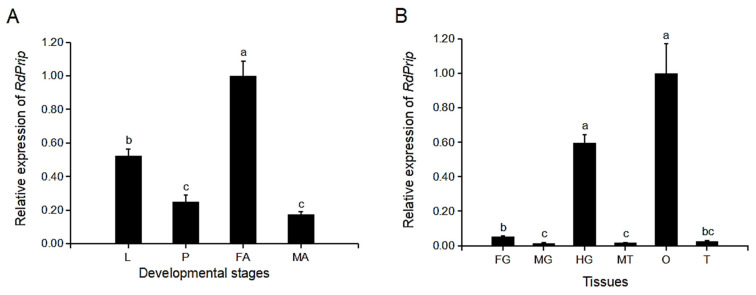
Developmental (**A**) and tissue (**B**) profiles of *RdPrip* expression. The error bar represents the standard error of three replicates. Different letters indicate significant differences between treatments (*p* < 0.05). L: larvae; P: pupae; FA: female adults; MA: male adults; FG: foregut; MG: midgut; HG: hindgut, including cryptonephridial systems; MT: Malpighian tubules; O: ovary; T: testis.

The developmental and tissue transcriptional expression profiles of *RdPrip* were determined using qPCR. *RdPrip* was expressed in all of the developmental stages tested. The expression was the highest in female adults, followed by larvae, pupae, and male adults. The differences in the expression levels of pupae and male adults were not statistically significant (Figure 2A). *RdPrip* expression in the ovary and the hindgut (including cryptonephridial systems) was significantly higher than that in the foregut, midgut, Malpighian tubules, and testis. The expression level was not significantly different between the ovary and hindgut, but it was 1.7-fold higher in the ovary than in the hindgut (Figure 2B).

### 3.3. Functional Studies of RdPrip

**Figure 3:**
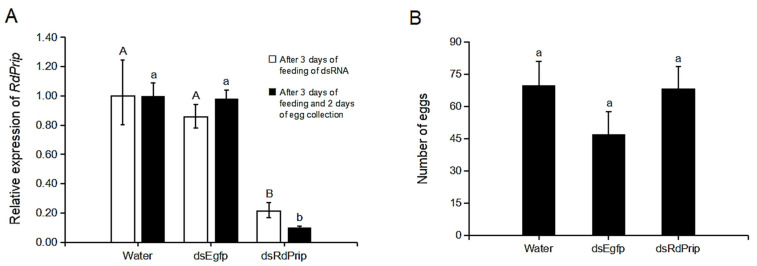

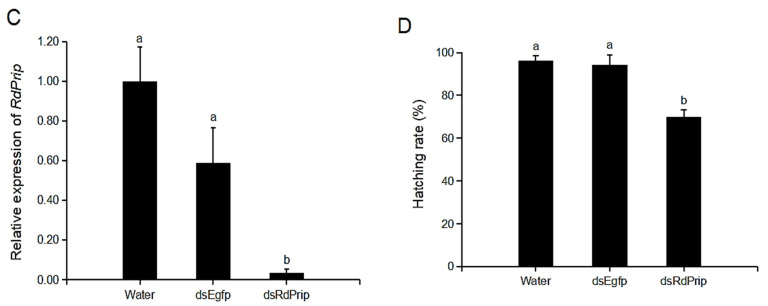
Functional studies of *RdPrip* on female reproduction. (**A**) The knockdown efficiency of *RdPrip* mRNA determined using qPCR. The uppercase and lowercase are for the comparisons among open and solid bars, respectively. (**B**) Fecundity of RNAi-treated female adults. (**C**) The transcriptional expression of *RdPrip* in eggs laid by RNAi-treated female adults. (**D**) The hatching rate of eggs laid by RNAi-treated female adults. The error bar represents the standard error of three replicates. Different letters indicate significant differences between treatments (*p* < 0.05).

Functional studies of *RdPrip* were performed using RNAi. After 3 days of feeding on ds*RdPrip* RNA, ds*Egfp* RNA, and water, the *RdPrip* transcriptional expression was decreased significantly, and the knockdown effect lasted for 2 days (Figure 3A). All of the females in the three treatment groups laid eggs, and the fecundity was not significantly different (Figure 3B). However, *RdPrip* expression was significantly lower in the eggs laid by the ds*RdPrip*-treated females than in those laid by females subjected to the two control treatments (Figure 3C). Furthermore, the hatching rate significantly decreased after *RdPrip* knockdown in female adults (Figure 3D).

### 3.4. Association of RdPrip Functions with Spinosad Resistance in R. dominica

**Figure 4:**
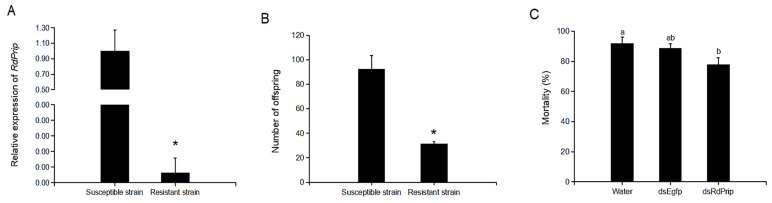
Association of *RdPrip* functions with spinosad in *R. dominica*. (**A**) qPCR analysis of *RdPrip* in susceptible and resistant strains. (**B**) The number of offspring was higher in susceptible strains than in resistant strains. (**C**) The mortality of *R. dominica* to spinosad exposure after knockdown of *RdPrip*. The error bar represents the standard error of replicates. * or different letters indicate significant differences between treatments (*p* < 0.05).

The relative transcriptional expression of *RdPrip* between susceptible and spinosad-resistant strains was detected using qPCR. The results revealed that the transcriptional expression was significantly lower in the resistant strain than in the susceptible strain (Figure 4A).

The lower *RdPrip* expression in resistant strains may result from lower levels of its expression in the ovary and/or the hindgut. To explore the association between *RdPrip* functions and resistance, the following two experiments were conducted. First, the offspring number of susceptible and resistant strains were compared, which revealed an average of 92.3 and 31.7 first-generation adults in susceptible and resistant strains, respectively, which was significantly different (Figure 4B). Second, the susceptibility of the *R. dominica* strain susceptible to spinosad was detected after *RdPrip* knockdown. The results revealed that mortality decreased after *RdPrip* knockdown. However, the mortality did not significantly differ between the ds*RdPrip* and ds*Egfp* treatment groups (Figure 4C).

## 4. Discussion

*R. dominica* is a major stored-product pest that feeds on dry grains in barns, where the humidity is low. Therefore, water homeostasis is critical in *R. dominica*. In this study, aquaporin prip (*RdPrip*) cDNA was cloned from *R. dominica*. The deduced amino acid sequence was almost identical to the one in the genome including all of the conserved domains of aquaporins (Figure 1).

The tissue distribution of *prip* mRNA varied in different insect species. The *prip* of *Tribolium castaneum* (*TcPrip*) is highly expressed in cryptonephridial systems, and its knockdown inhibits water reabsorption, resulting in more water loss and leading to the reduction of larval and pupal mass [32]. The expression levels of *prip* of *Chilo suppressalis* (*CsPrip*) were reported to be comparable among the Malpighian tubules, fat body, hindgut, head, and epidermis, which suggests the multiple functions of *CsPrip* in maintaining water homeostasis [33]. In *Blattella germanica*, *prip* expression is the highest in the ovaries, followed by the fat body and muscles, and it is almost undetectable in the digestive tract, Malpighian tubules, and colleterial gland [12]. The high *RdPrip* expression in the ovary may contribute to the highest transcriptional expression of *RdPrip* in female adults in this study (Figure 2).

The finding of high *RdPrip* expression in the ovaries and female adults suggests its potential role in reproduction (Figure 2). In *Apis mellifera*, six aquaporin genes are expressed in the ovaries of both virgin and mated queens. The expression level of *Am-Eglp2* is significantly high in mated and 4-day-old virgin queens. Moreover, *Am-Prip* is highly expressed in 4-day-old virgin queens, which is the time expected to mate. In addition, another aquaporin, drip, was immunolocalized in the follicular cells of the mated queen. These results indicate that aquaporins are involved in honeybee reproduction [15]. In *B. mori*, there are two water-specific aquaporins, drip and prip, present at different ovarian development phases and they play different roles in ovarian maturation [14]. The *Bm*Drip protein is abundantly expressed in the yolk granules under the oocyte membrane. The distribution of *Bm*Drip is inferred to avoid water evaporation. The distribution of *BmPrip* is at the oocyte plasma membrane and transports water during vitellogenesis for the hydration and swelling of oocytes [14]. This swelling phenomenon has also been reported in *D. melanogaster*. Prip is the only aquaporin in *Drosophila* ovaries, and it regulates water transport in the ovaries to result in swelling, which is essential for calcium propagation and to initiate egg activation, thereby initiating the embryogenesis of mature oocytes [34]. In addition to prip, there are other aquaporins found in insect ovaries. The aquaporin bib of the bed bug, *Cimex lectularius* (*ClBib*), is highly expressed in ovaries and female adults, and its knockdown increases the fecundity of bed bugs, suggesting a role in reproduction inhibition in this species [35]. In this study, *RdPrip* knockdown in *R. dominica* females did not affect the number of eggs laid (Figure 3B) but decreased their hatching rate (Figure 3D). In addition, *RdPrip* expression was low in the eggs laid by *dsRdPrip*-treated females (Figure 3C). On the basis of these results, we suggest that *RdPrip* plays a vital role in embryogenesis, not in oogenesis. This study is the first to describe the functional role of aquaporin prip in the reproduction in coleopterans.

*RdPrip* expression and the number of offspring were significantly lower in the spinosad-resistant strain than in the susceptible strain (Figure 4A,B). The decreased offspring number may be associated with reduced *RdPrip* expression, or other fitness costs. In *Anopheles coluzzi* and *Frankliniella occidentalis*, the expression levels of aquaporins are higher in insecticide-resistant populations [36,37]. This is contrary to our result, but they do not demonstrate in which tissue the aquaporins are expressed. However, the highly expressed aquaporins in resistant *A. gambiae* Malpighian tubules could be related to the excretion of insecticide [10,38]. The function of Malpighian tubules in insect excretion is to secrete water. On the other hand, the function of the hindgut is to reabsorb water. In the hindgut (including cryptonephridial systems), the lower the *RdPrip* expression, the less the water reabsorption. Therefore, more water would be eliminated with the excreta, including the metabolized pesticide, to increase the resistance. *RdPrip* knockdown in the susceptible strain to mimic the resistant strain did not significantly lower the susceptibility of *R. dominica* to spinosad (Figure 4C). This suggests the presence of other aquaporins in the hindgut and indicated that functionally redundant aquaporins can compensate for the reabsorption function of *RdPrip*.

In this study, we constructed a useful feeding methodology to knockdown gene expression in coleopterans. Injecting small insects such as *R. dominica* adults (3–4 mm) is difficult. Feeding methodology enables the convenient application of RNAi to a large number of insects at once. Our results revealed that *RdPrip* was highly expressed in ovaries, and knockdown of *RdPrip* reduced the number of offspring. In addition, knockdown of *RdPrip* would not only reduce the susceptibility to spinosad but, perhaps, other insecticides. Therefore, *RdPrip* should be a useful candidate for *R. dominica* control whether or not combined with insecticides.

## Figures and Tables

**Table 1:** Sequences of primers used in this study.

Primer Name	Primer Sequence
RdDripdf	GGAKGHCAYRTHAAYCCSGCKGTRAC
RdDripdr	ABBGGWCCMRCCCARTAMAYCCAWTG
RdDrip450-1	ATCAAGGGACTGCTATACGTCGTCG
RdDrip450-2	CATTGTCCTGCGATCAAAGCAGACC
RdDrip450-3	TACAATGTCTGGGAGCATTAGCTGG
RdDrip450-4	TGTAGTCTACAGCCGCCAAATGTCC
RdPrip1008-1	GCGATTCCTACAGTACAGTTCGGTTACG
RdPrip1008-2	ATGTAGGCATTAGATGAGTGTTGTGCGC
RdPrip qPCR-1	GGCAATCTAGTTGTAGGTAATATAC
RdPrip qPCR-2	CTCAGTCAAACCAGAATCG
Rd-18s qPCR-01	CGAGACTCTGGCCTGCTAAC
Rd-18s qPCR-02	CCGCCTGTCCCTCTAAGAA
RdPripdsRNA-03	TAATACGACTCACTATAGGGAGATTCTGGTTTGACTGAGCTGCAAG
RdPripdsRNA-04	TAATACGACTCACTATAGGGAGAGCATTAGATGAGTGTTGTGCGCT

## Data Availability

Data are contained within the article.
